# Extraction and Optical Analysis of the Extracellular Fluid from the Body Segments of *Apis mellifera* Bees

**DOI:** 10.1093/iob/obaf018

**Published:** 2025-05-09

**Authors:** J Hernández, F Mesa, A Riveros, R Fayad, J Nisperuza

**Affiliations:** Departamento de Biología, Facultad de Ciencias Naturales, Universidad del Rosario, Bogotá, Colombia; NanoTech Group, Facultad de Ingeniería y Ciencias Básicas, Fundación Universitaria Los, Libertadores, Cra 16 No. 63a-68, Bogotá 111221, Colombia; Departamento de Biología, Facultad de Ciencias Naturales, Universidad del Rosario, Bogotá, Colombia; Departamento de Biología, Facultad de Ciencias Naturales, Universidad del Rosario, Bogotá, Colombia; NanoTech Group, Facultad de Ingeniería y Ciencias Básicas, Fundación Universitaria Los, Libertadores, Cra 16 No. 63a-68, Bogotá 111221, Colombia

## Abstract

This study developed and optimized a methodology based on controlled centrifugation for the segmented extraction of extracellular fluid in *Apis mellifera* bees. Three critical variables were analyzed: relative centrifugal force, centrifugation time, and the number of individuals processed, ensuring the reproducibility and efficiency of the procedure. The results demonstrated significant differences in the volume of fluid recovered from different body segments, with the abdomen yielding the highest volumes, followed by the thorax and the head. UV-Vis spectroscopic characterization revealed distinct optical features for the samples, identifying specific absorbance peaks unique to each segment. Furthermore, biochemical analysis using Benedict's reagent confirmed the presence of reducing sugars, with head samples displaying the most intense coloration. These findings underscore the importance of segment-specific analysis to gain deeper insights into the physiology and metabolism of bees. The proposed methodology offers a novel and robust tool for physiological, metabolic, and ecotoxicological studies, facilitating the assessment of environmental and contaminant impacts on pollinator health.

## Introduction

The extracellular fluid in insects plays essential roles in the transport of nutrients, hormones, and metabolites (Chen et al. 2021; Strachecka et al. 2022; Elfar et al. 2023a; Tafi et al. 2024; Wang et al. 2021), as well as in osmotic regulation (Nakamatsu and Tanaka 2004; Cao et al. 2020; Holdbrook et al. 2024) and the response to stress agents (Tauchman et al. 2007; Lubawy and Słocińska 2020). In *Apis mellifera* bees, studying this fluid is key to assessing their physiological and metabolic state, especially under environmental stress conditions and contaminant exposure (Brandt et al. 2016; Kim et al. 2022). However, existing methodologies for its extraction have significant limitations regarding volume control, reproducibility, and regional resolution.

Traditional methods for fluid extraction in insects, such as using hypodermic needles or glass capillaries, focus on globally and uniformly extracting hemolymph (Borsuk et al. 2017; Butolo et al. 2020). These approaches assume that the extracellular fluid is homogeneously distributed throughout the insect's body, overlooking physiological and metabolic differences among body segments, such as the head, thorax, and abdomen. Previous studies have demonstrated that these regions have specialized energy demands and functions. For instance, the abdomen stores large quantities of liquid and energy reserves, whereas the thorax, dominated by muscle tissue, exhibits highly active metabolism to support flight (Blow and Douglas 2019; Chang et al. 2023).

Recently, centrifugation-based methods have been used as an alternative for efficient extracellular fluid extraction in insects (Tabunoki et al. 2019; Niu et al. 2023). However, these approaches have not considered the segmented extraction of body parts or systematically evaluated physical parameters such as angular velocity, centrifugation time, and the number of individuals processed. The segmented extraction of extracellular fluid by body regions is essential to obtain detailed information on regional metabolic processes, enabling more precise comparative analyses.

This study aimed to develop and optimize an efficient method for the segmented extraction of extracellular fluid in *Apis mellifera* bees using controlled centrifugation. Additionally, the extracted samples were optically characterized using UV-Vis spectroscopy to detect optical absorbances (Schenk et al. 2015; Park et al. 2017; Ushakova et al. 2019). Extractions were also conducted to identify the presence of reducing sugars using Benedict's reagent, as previously performed in studies with insects and food samples (Park et al. 2017; Ratanawimarnwong et al. 2022). The centrifugation method provides control over the volume and localization of the extracted fluid, offering a robust tool for physiological (Bogaerts et al. 2009; Elfar et al. 2023a; Kannan et al. 2024; Li et al. 2024), metabolic (Cournoyer et al. 2022; Chang et al. 2023; Wang et al. 2021), ecotoxicological (Paleolog et al. 2020; Migdał et al. 2021), and comparative studies in insects (Chan et al. 2006; Elfar et al. 2023b).

Traditional hemolymph extraction techniques, such as puncturing the cuticle with glass capillaries or hypodermic needles, generally produce pooled fluid from various tissues, limiting the resolution of region-specific physiological information. The method proposed here enables segment-specific sampling through centrifugation, offering the possibility of detecting localized metabolic and physiological variations that remain masked by conventional approaches (Borsuk et al. 2017; Butolo et al. 2020). While previous centrifugation-based strategies have demonstrated high efficiency in extracting hemolymph in other insect species, they have excluded both segmental separation and systematic evaluation of physical variables such as angular velocity, extraction time, and number of individuals processed (Tabunoki et al. 2019; Niu et al. 2023).

The current methodology incorporates moderate centrifugal forces and low temperatures to preserve tissue integrity and promote the recovery of extracellular fluid. The design avoids the use of sonication, chemical lysis, or mechanical disruption, which helps maintain cell structure throughout the extraction process. The analytical scope focused on optical and chromatic characterization, and the segment-dependent consistency of the results supports the interpretation that the recovered fluid predominantly reflects extracellular composition. Additional considerations regarding fluid purity and possible intracellular contributions are addressed in the discussion.

## Materials and methods

### Experimental animals

**Fig. 1:**
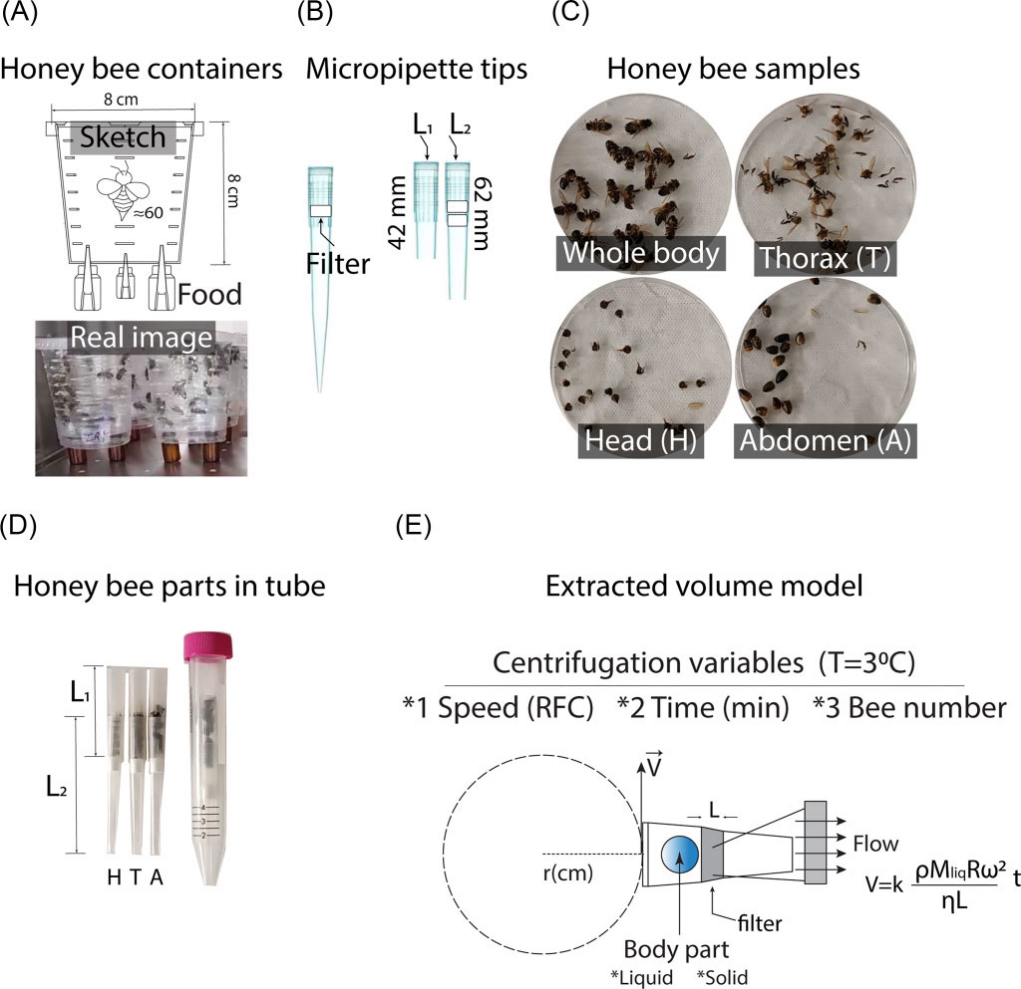
Experimental setup for bee maintenance and extracellular fluid extraction. (A) Bees in containers with feeders. (B) Modified pipette tips. (C) Dissection of insects and final assembly in conical tubes. (D) Model of the extraction process.

The Africanized *Apis mellifera* bees used in this study were collected from the apiary at the Universidad del Rosario (Bogotá, Colombia). Collection was conducted during the early morning hours to minimize thermal stress and ensure that the insects were in optimal physiological condition (Abou-Shaara et al. 2012; Butolo et al. 2021; Stupski and Schilder 2021). Bees were manually captured using plastic containers (disposable cups) with ventilation slots to allow respiration, as recommended in various protocols (Köhler et al. 2013; Williams et al. 2013; Huang et al. 2014). Immediately after collection, they were transported to the laboratory, where they were kept under controlled temperature conditions (35 ± 3°C), constant relative humidity (60 ± 5%), and specific light wavelengths (730 nm) to avoid stress caused by the presence of the experimenter (Dexheimer et al. 2022; Somanathan 2024; Wang et al. 2022). The bees were fed ad libitum with a 1 M sucrose solution (31.1% w/w) provided through feeders (Fig. 1A).

In all experiments, the bees were euthanized by exposure to a temperature of −30°C in a cooling unit (Thermo Scientific Revco Freezer UGL3020A), where they remained for 24 h. The bees were then prepared for the experiments, (1) body mass measurement, (2) extracellular fluid extraction, (3) UV-Vis spectroscopic characterization, and (4) sugar detection. All activities involving the use and handling of bees were carried out in compliance with ethical standards for working with bees ensuring conditions that minimized stress to the individuals.

### Experimental setup for extracellular fluid extraction

The experimental setup for extracting extracellular fluid was designed using common laboratory materials, such as pipette tips and conical centrifuge tubes. The pipette tips used were 1000 µL tips with a 0.2 µm filter (pore size), and the tubes had a capacity of 15 mL. The narrow ends of the tips were cut, resulting in lengths of L1 = 42.0 ± 0.1 mm and L2 = 62.0 ± 0.1 mm. The filter from L1 was removed and inserted into L2, and the final tip (L) was obtained by nesting L1 into L2 (Fig. 1B). The bees were dissected into 3 segments (head, thorax, and abdomen), which were placed into the final tip (L), and this system was then inserted into the centrifuge tubes. Each segment was supplemented with 500 µL of distilled water to preserve the extracted extracellular fluid and facilitate sample flow (Fig. 1C). Finally, the setup was placed in a centrifuge (Thermo Scientific ST 16R; rot: 75003181) programmed with a constant temperature (3°C), angular velocity (relative centrifugal force [RCF]), and time (min).

The extraction of extracellular fluid was based on the centrifugal force applied to the body segments:


(1) \begin{equation*}
{F}_c = {\mathrm{MR}}{\omega }^2,
\end{equation*}

where M is the mass of the body segment, R represents the effective centrifugation radius, and ω is the angular velocity. The volume of extracellular fluid extracted (V_ext_) was estimated considering the pressure difference generated during centrifugation, following the equation:


(2) \begin{equation*}
{V}_{ext} = \frac{{k\rho {M}_{liq}R{\omega }^2}}{{\eta L}}t,
\end{equation*}

where *k* is the system's proportionality constant, $\rho $ is the fluid density, corresponding to the mass of the extracted fluid, η is the fluid's viscosity, *L* is the effective length of the filter, and *t* is the centrifugation time (Fig. 1D).

These equations provide the theoretical foundation for the extraction system. The centrifugal force formula illustrates how differences in segment mass and angular velocity influence the mechanical stress applied during centrifugation. The second expression outlines how fluid extraction depends on pressure gradients, viscosity, and filter geometry. Although no direct calculations were performed using these equations, they serve to conceptually support the design of the extraction setup and the interpretation of experimental trends across segments and conditions.

At the end of the centrifugation process, the tips were carefully removed from the conical tubes, and the recovered extracellular fluid was transferred to Eppendorf tubes for storage at −20°C. The samples obtained were subsequently used for optical analysis through UV-Vis spectroscopy and Benedict's reagent tests.

### Experiment 1: Distribution of body mass and water content in *Apis mellifera*

The first experiment aimed to characterize the distribution of body mass and water content in *Apis mellifera*, both at the whole-body level and within its main body segments (head, thorax, and abdomen). This experiment included 3 main evaluations, (1) body mass distribution; (2) water content in the whole body; and (3) water content in the body segments. These evaluations sought to address questions related to the distribution of body mass, water content, and its allocation among body segments.

### Body mass distribution

To determine the distribution of body mass, the total mass of individual bees was measured by using an analytical balance with a precision of 0.1 mg. Subsequently, each bee was dissected into its main body segments: head, thorax, and abdomen. The mass of each segment was recorded individually immediately after dissection to prevent fluid loss due to evaporation. This data allowed for the calculation of mass distribution within the bee's body, providing a detailed characterization of the proportion of mass attributed to each segment.

### Water content in the body of bees

The water content in the body was determined by measuring the wet and dry mass. Initially, the mass of each bee was measured immediately after capture, referred to as the wet mass (Wet). Subsequently, the insects were dried in an oven at 70°C for 3 days to remove all body water. At the end of this process, the dry mass (Dry) was measured. The body water content (Water) was calculated as the difference between the wet and dry mass (Water = Wet − Dry). Additionally, a linear regression analysis was performed between Water and Wet to develop a model describing the relationship between water content and total body mass. The slope of the model was used as the body water mass factor.

### Water content in body segments

The bees were dissected into their main body segments (head, thorax, and abdomen) to evaluate the water content in each segment. The previously described protocol for measuring wet and dry mass was applied to each segment. The data obtained provided a precise reference for the distribution of water among the body segments, offering essential information for interpreting the results of extracellular fluid extraction.

### Experiment 2: Extracellular fluid extraction from body segments using centrifugation

The extraction of extracellular fluid in *Apis mellifera* was conducted using centrifugation methods for each body segment. The experiment was designed to evaluate the variables of angular velocity, centrifugation time, and the number of insects. These evaluations aimed to address questions regarding the amount of extracellular fluid that could be obtained with this method and how centrifugation variables influence the extraction process.

The extraction process was evaluated using 3 variables: angular speed (Speed), centrifugation time (Time), and the number of bees (#Bees). The independent variables were tested at 5 levels: Speed (RCF) = 400,1000, 1600,2200, 2900; Time (min) = 2, 4, 6, 8, 10; #Bees = 2, 4, 6, 8, 10. The extraction process was designed to vary one of the 3 variables at a time while keeping the other two constant. The final evaluation of all variable changes was designed to converge at the following values: Speed = 2900 RCF; Time = 10 min; #Bees = 10 bees. The quantification of the extracted extracellular fluid was performed by measuring the mass of the samples using a precision balance (Radwag AS 220.R2) before and after centrifugation. The extracted mass was determined by the difference between the two measurements, with 5 repetitions conducted for each evaluation.

For each condition, segments from multiple bees (2, 4, 6, 8, or 10) were pooled and centrifuged together as a single sample. The mass of extracted fluid was calculated as the difference between the total mass of the container system before and after centrifugation, allowing quantification of the fluid derived exclusively from the insect tissue. Although 500 µL of distilled water was added before centrifugation to preserve fluidity and minimize sample loss, this added water was not included in the extracted mass, as it remained in the upper portion of the container and did not interfere with the measurement of the recovered fluid.

### Experiment 3: UV-Vis spectroscopic characterization of extracellular fluid from body segments

The characterization was performed on the extracellular fluid extracted from the body segments of bees, which were processed through centrifugation at 2000 RCF for 4 min; this process was repeated 10 times. The fluid samples were analyzed using UV-Vis spectroscopy to explore differences among the samples.

The experiment was designed to evaluate the absorbance of the segments (head, thorax, and abdomen) across a wavelength interval of 200–590 nm, utilizing a small-sample spectrophotometer (Thermo Scientific NanoDrop 2000). This experiment aimed to address questions related to the optical absorbance of extracellular fluid based on body segments, as well as to investigate differences among the segment-derived samples.

### Experiment 4: Sugar test for extracellular fluid using Benedict's reagent

The reduced sugar test was conducted on extracellular fluid samples extracted using the centrifugation method (Bees = 5; ω = 2000 RCF; *t* = 4 min); the extractions were repeated 16 times. In test tubes, extracellular fluid samples (0.5 mL) were mixed with Benedict's reagent (30 mL). The tubes were placed in a beaker containing 300 mL of boiling water for 10 min. After removing the boiling water, the samples were allowed to cool to room temperature for 20 min, after which photographs of the samples were taken. Six samples of Benedict's reagent served as the control.

All photographs were taken under standardized conditions to ensure consistency in red, green, and blue (RGB) analysis. Samples were placed on a white matte background and illuminated with uniform white LED lighting (6000 K). Images were captured using a Xiaomi 12 Pro smartphone, mounted on a fixed support 20 cm above the samples. The same shooting mode, focus, and exposure settings were used for all photographs. No filters or digital enhancements were applied. RGB values were extracted using Fiji (ImageJ) under identical image-processing conditions for all samples.

The evaluation of the color changes in the mixtures was performed using Fiji (ImageJ) software, which quantified the RGB color components in the photographs. The experiment aimed to address questions regarding the presence of reducing sugars in the samples and potential variations in coloration depending on the body segment from which the fluid was extracted.

## Results

### Experiment 1: Distribution of body mass and water content in *Apis mellifera*

#### Body mass distribution

**Fig. 2:**
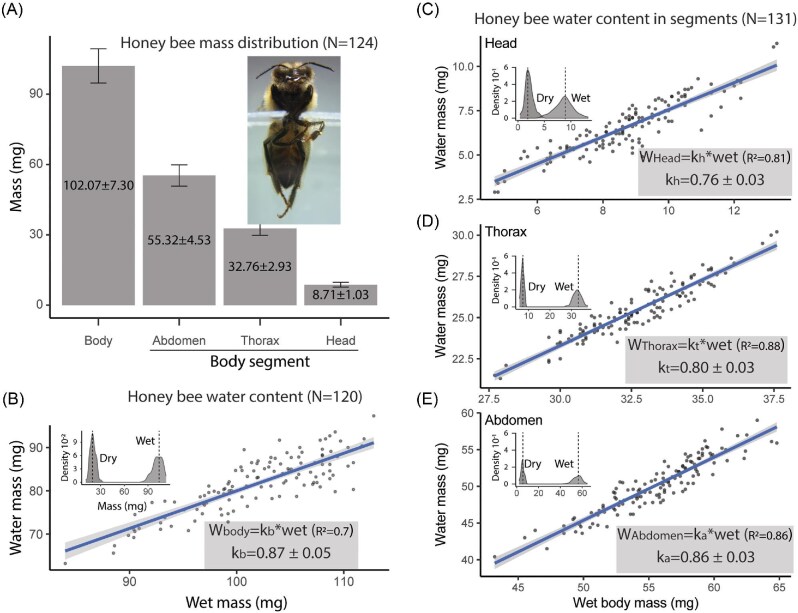
Distribution of body mass and water content in *Apis mellifera*. (A) Body mass distribution. (B) Whole-body water content, showing the relationship between wet mass and dry mass. (C, D, E) Water content distribution across body segments.

A total of 124 bees were analyzed to determine the distribution of body mass across the whole body and its main segments (head, thorax, and abdomen). The average total body mass was 102.07 ± 7.30 mg. Among the body segments, the abdomen contributed the most to the total body mass, with an average of 55.23 ± 4.53 mg, followed by the thorax (32.76 ± 2.93 mg) and the head (8.71 ± 1.03 mg). These results indicate that the abdomen accounts for more than half of the total body mass of *Apis mellifera*, while the head contributes less than 10%. The body mass distribution provides a clear reference for analyses related to extracellular fluid extraction (Fig. 2A).

#### Water content in the body of bees

The estimation of body water content was performed on 120 bees by measuring their wet mass and dry mass. The average wet mass was 101.92 ± 6.07 mg, while the average dry mass was 20.26 ± 3.54 mg. Based on these measurements, the body water content (Water) was calculated as the difference between wet and dry mass, resulting in an average of 81.66 ± 6.30 mg. Furthermore, a linear regression analysis between Water and Wet revealed a significant slope (*k*_b_ = 0.87 ± 0.05; *R*² = 0.7; *P* < 0.001), indicating that 87% of the wet mass corresponds to water content (Fig. 2B). These findings emphasize that most of the body mass in bees is composed of water, a critical factor for extracellular fluid extraction analyses.

#### Water content in body segments

The analysis of water content in body segments (head, thorax, and abdomen) was conducted on a total of 131 bees. The results indicated that the abdomen contains the highest water content, with an average of 50.1 ± 3.6 mg, followed by the thorax (25.4 ± 1.6 mg) and the head (6.6 ± 1.5 mg). The wet and dry masses of each segment were also measured, providing a detailed reference for relative proportions.

Regression analysis revealed a significant relationship between Water and Wet for all segments, with slopes of *k*_H_ = 0.76 ± 0.03 (*R*²=0.81) for the head, *k*_T _= 0.80 ± 0.03 (*R*²=0.88) for the thorax, and *k*_A _= 0.86 ± 0.03 (*R*²=0.86) for the abdomen. These results demonstrate that water content relative to wet mass varies slightly between segments, with the abdomen showing the highest percentage of water (Fig. 2C, D, and E); this distribution is crucial for interpreting extracellular fluid extraction patterns in each body segment.

### Experiment 2: Extracellular fluid extraction from body segments using centrifugation

#### Fluid mass extracted from the head segment

To calculate the percentage of extracted fluid, we measured the total mass of the collection system (segment plus container) before and after centrifugation, subtracting the prerecorded mass of the empty container. The percentage of fluid recovered was then determined as (final mass/initial mass) × 100. This approach provided a relative measure of extraction efficiency for each condition tested.

Segmental reference masses and water content values from Experiment 1 were used to interpret these percentages and contextualize extraction dynamics. Although we referred to “proportional extraction” to indicate that fluid recovery increased with centrifugation time, force, or number of bees, the trends were not strictly linear. Instead, extraction curves showed positive but saturating behaviors, consistent with physical limitations on fluid displacement under centrifugation.

#### Fluid mass extracted from the head segment

**Fig. 3:**
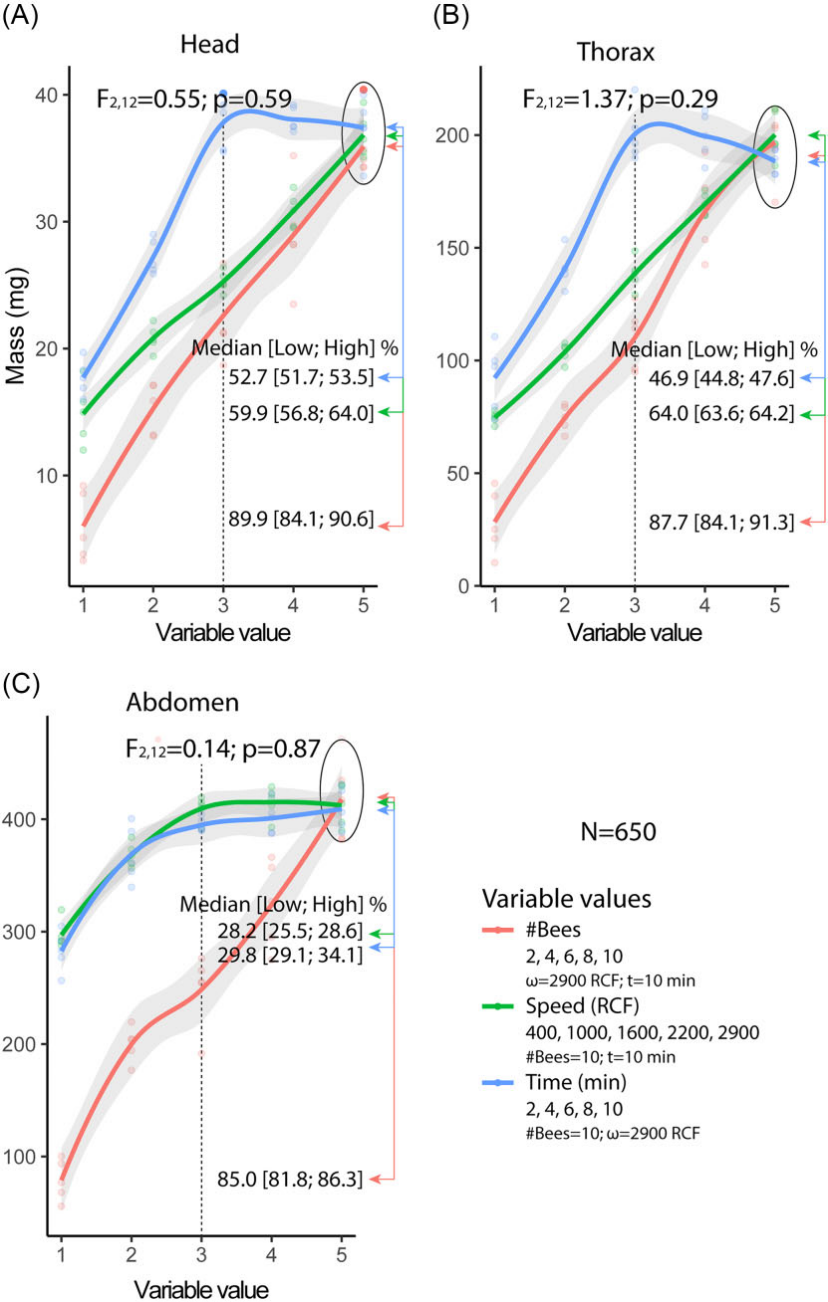
Effect of independent variables on the extraction of extracellular fluid in *Apis mellifera*. (A) Volume of extracellular fluid extracted from the head segment. (B) Volume extracted from the thorax segment. (C) Volume extracted from the abdomen segment.

The independent variables (#Bees, Speed, and Time) significantly influenced the extraction of extracellular fluid from the head segment (*F*₂,₇₂=7.31, *P* < 0.001; η²=0.169). However, under the maximum variable conditions, the differences were not significant (*F*₂,₁₂=1.58, *P* = 0.25). Increases in the number of bees (Speed = 2900RCF; Time = 10 min) led to progressive increases in fluid recovery, with the percentage of initial to final measurements exceeding 80% (median, IQR [Low; High] %): 89.9, IQR [84.1; 90.6]. Variations in Speed (#Bees = 10; Time = 10 min) showed a trend of increasing extraction that stabilized at higher speeds, with percentages above 56% (median %, IQR [Low; High] %): 59.9, IQR [56.8; 64.0]. Finally, changes in Time (Bees = 10; Speed = 2900 RCF) resulted in increased recovery during the first intervals, followed by a plateau, with percentages exceeding 51% (median %, IQR [Low; High] %): 52.7, IQR [51.7; 53.5] (Fig. 3A). These results were interpreted in light of the segmental mass and water content data obtained in Experiment 1, which provided a reference for estimating the expected amount of fluid per segment.

#### Fluid mass extracted from the thorax segment

The independent variables (#Bees, Speed, and Time) significantly influenced the extraction of extracellular fluid from the thorax segment (*F*₂,₇₂=5.61; *P* < 0.001; η²=0.135). However, under the maximum variable conditions, the differences were not significant (*F*₂,₁₂=0.81; *P* = 0.47). Increases in the number of bees (Speed = 2900 RCF; Time = 10 min) produced progressively higher extraction values with a trend toward saturation, with the percentage of initial to final measurements exceeding 84% (median, IQR [Low; High] %): 87.7, IQR [84.1; 91.3]. Variations in Speed (#Bees = 10; Time = 10 min) yielded consistently elevated values, with percentages above 63% (median %, IQR [Low; High] %): 64.0, IQR [63.6; 64.2]. Finally, changes in Time (#Bees = 10; Speed = 2900 RCF) showed increased recovery during the first intervals, followed by a plateau, with percentages exceeding 44% (median %, IQR [Low; High] %): 46.9, IQR [44.8; 47.6] (Fig. 3B).

#### Fluid mass extracted from the abdomen segment

The independent variables (#Bees, Speed, and Time) significantly influenced the extraction of extracellular fluid from the abdomen segment (*F*₂;₇₂=19.550; *P* < 0.0001; η²=0.352). However, under the maximum variable conditions, the differences were not significant (*F*₂;₁₂=1.45; *P* = 0.27). Increases in the number of bees (Speed = 2900 RCF; Time = 10 min) led to a strong increase in fluid recovery, with the percentage of initial to final measurements exceeding 81% (median, IQR [Low; High] %): 85.0, IQR [81.8; 86.3]. Variations in Speed (#Bees = 10; Time = 10 min) resulted in nonlinear recovery trends, with the highest yields at lower speeds, and the percentage of initial to final measurements exceeding 25% (median %, IQR [Low; High] %): 28.2, IQR [25.5; 28.6]. Finally, changes in Time (#Bees = 10; Speed = 2900 RCF) mirrored the speed condition trend, with percentages exceeding 29% (median %, IQR [Low; High] %): 29.8, IQR [29.1; 34.1] (Fig. 3C). As with the other segments, interpretation was supported by segmental mass data obtained from Experiment 1.

Under optimized conditions (2900 RCF, 10 min, 10 bees), the average fluid mass extracted per segment was approximately 1.3 mg for the head, 3.2 mg for the thorax, and 7.5 mg for the abdomen. Assuming a fluid density close to that of water (1 g/mL), these values correspond to volumes of approximately 1.3 µL, 3.2 µL, and 7.5 µL, respectively, for each 10-bee sample. On a per-bee basis, this represents an average recovery of ∼0.13 µL for the head, ∼0.32 µL for the thorax, and ∼0.75 µL for the abdomen.

### Experiment 3: UV-Vis spectroscopic characterization of extracellular fluid from body segments

**Fig. 4:**
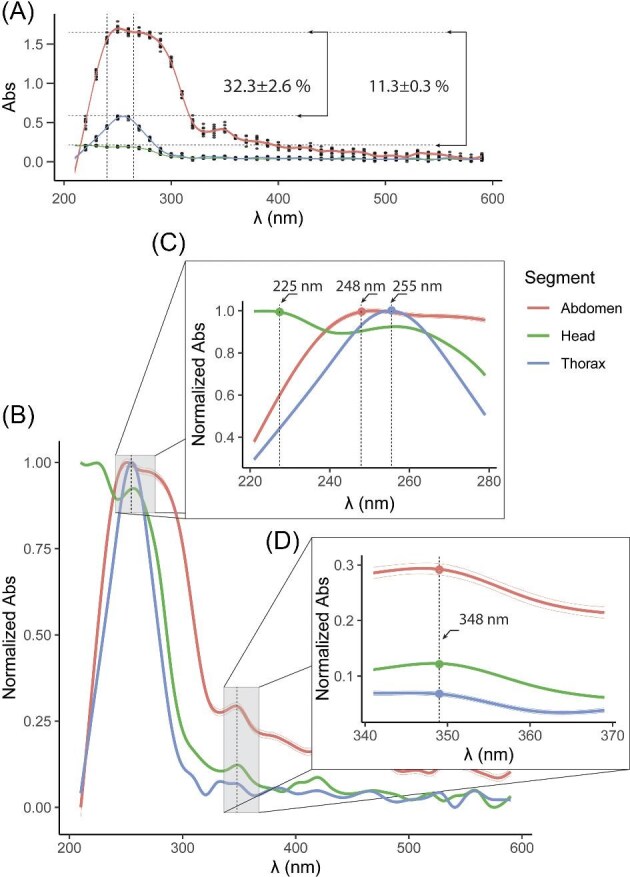
Optical spectroscopy curves for extracellular fluid samples by body segment. (A) Absorbance curves for the head, thorax, and abdomen segments. The curve maxima are in the 220–260 nm range. The absorbance values for the head and thorax are below the curve of the abdomen, with percentages of 11.3% and 32.3%, respectively. (B) Normalized absorbance curves for the body segments. (C) Wavelengths defining the maximum of normalized absorbance. (D) Wavelength defining local maxima of normalized absorbance in the 340–370 nm range.

The absorbance of the extracellular fluid showed maximum values between 220 and 260 nm, which were highest for the abdomen, intermediate for the thorax, and lowest for the head (Abs = mean ± SD): 0.189 ± 0.002 (Head); 0.540 ± 0.042 (Thorax); 1.671 ± 0.032 (Abdomen). These results indicate that absorbance values depend on the body segment. Relative to the abdomen, the absorbance percentage for the thorax and head was below 32% (mean ± SD %): 32.3 ± 2.6 (Thorax/Abdomen); 11.3 ± 0.3 (Head/Abdomen); this suggests that absorbance varied according to the amount of extracellular fluid extracted, with the abdomen having the highest values, the thorax intermediate, and the head the lowest (Fig. 4A).

Normalized absorbance trends showed significant differences (*F*₂_;_₁₁₄₀ = 28.71, *P* < 0.0001) across the 210–590 nm wavelength range. Significant differences (*P* < 0.001) were also observed between pairs of segments (Diff; CI [low; high]): −0.092; CI [−0.144, −0.041] (Abdomen-Head); −0.167; CI [−0.219, −0.115] (Abdomen-Thorax); −0.075; CI [−0.126, −0.023] (Head-Thorax) (Fig. 4B). The wavelengths corresponding to the maximum normalized absorbance values occurred in different regions of the spectrum depending on the body segment: 224 nm (Head); 248 nm (Abdomen); 255 nm (Thorax) (Fig. 4C). Additionally, despite significant differences between the segments, the 348 nm wavelength defined 3 local maxima within the 340–370 nm range (Fig. 4D). These findings demonstrate that the extracellular fluid varies according to the body segment it originates from; however, some regions of the spectrum exhibit similarities.

### Experiment 4: Sugar test for extracellular fluid using Benedict's reagent

**Fig. 5:**
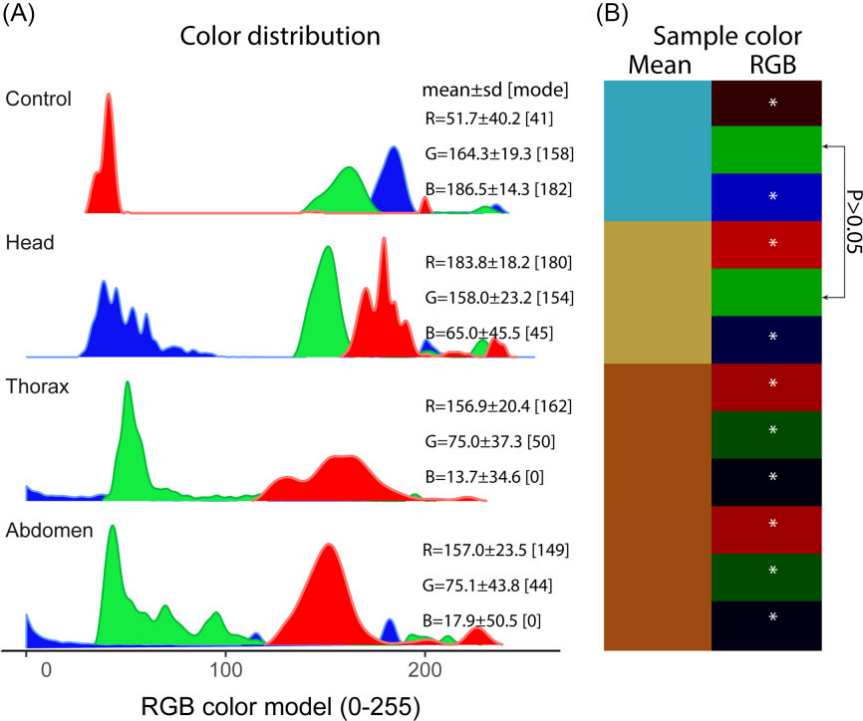
Coloration of body segment samples mixed with Benedict's reagent for sugar detection. (A) Distribution of sample coloration in RGB (0–255). (B) Average combination of RGB colors to produce the coloration of the samples with the reagent.

The RGB colors exhibited by the samples demonstrated the presence of traces of reducing sugars, as the coloration deviated from the blue of the reagent. Samples from the segments reacted with the reagent, displaying reddish coloration (mean ± SD) [mode]: 193.8 ± 18.2 [180] (Head); 156.9 ± 20.4 [162] (Thorax); 157.0 ± 23.5 [149] (Abdomen). Samples from the thorax and abdomen were characterized by low levels of green coloration compared to the head (mean ± SD) [mode]: 75.0 ± 37.3 [50] (Thorax); 75.1 ± 43.8 [44] (Abdomen); 158.0 ± 23.1 [154] (Head). The blue levels for all segments were the lowest (mean ± SD) [mode]: 65.0 ± 45.5 [45] (Head); 13.7 ± 34.6 [0] (Thorax); 17.9 ± 50.5 [0] (Abdomen) (Fig. 5A).

The coloration of the samples showed significant differences (*P* < 0.0001), indicating that the characteristics of the extracellular fluid depend on the body segment (*F*₃_;_₁₃₇₇₄ = 472.5). Each RGB component exhibited significant differences (*P* < 0.0001) across all samples: *F*₃_;_₅₀₆₀=788.3 (Blue); *F*₃_;_₅₀₃₈=707.6 (Green); *F*₃_;_₃₆₆₈=724.4 (Red). However, the green coloration for head samples showed no significant differences compared to Benedict's reagent (Diff; CI [Low; High]; *P*): = −1.38; CI [−7.01; 4.31]; 0.925. On the other hand, although the thorax and abdomen samples appeared visually similar, their defining RGB components showed significant differences (Fig. 5B). These results demonstrate that the coloration of the samples depended on the body segment from which they were extracted.

## Discussion

This study developed and validated a methodology based on segmented centrifugation for the extraction of extracellular fluid in *Apis mellifera* bees. The results demonstrated that the technique allows for the recovery of significant and reproducible volumes of extracellular fluid, highlighting important differences among body segments (head, thorax, and abdomen). The proposed methodology constitutes a robust and innovative tool for physiological, metabolic, and ecotoxicological studies in insects, addressing the limitations of traditional hemolymph extraction methods.

### Importance and relevance of the methodology

The implementation of the dual-filter system and the optimization of centrifugation variables allowed for the standardization of extracellular fluid extraction. Unlike conventional methods that often rely on hypodermic needles or glass capillaries (Borsuk et al. 2017; Butolo et al. 2020), this technique ensures cleaner sample handling, reduces the risk of tissue contamination, and facilitates the simultaneous processing of multiple individuals. Moreover, the use of modified pipette tips ensures efficient retention of body segments, enabling rapid and controlled separation of extracellular fluid.

The results showed that the abdomen releases larger volumes of extracellular fluid, consistent with its role as the main region for hemolymph storage (Blow and Douglas 2019). In contrast, the head and thorax exhibited lower volumes, likely due to dense tissue composition and a relatively smaller amount of extracellular fluid; these findings underscore the necessity of analyzing each segment separately, as global methods may obscure critical physiological differences.

These segmental fluid volumes are comparable to or greater than those obtained through traditional hemolymph collection methods, which typically yield 1–5 µL per bee using glass capillaries or hypodermic needles (Borsuk et al. 2017; Butolo et al. 2020). In contrast, our centrifugation-based approach enables simultaneous extraction from multiple individuals while providing segment-specific resolution and minimizing tissue damage or contamination.

Another consideration relates to the possible contamination of abdominal fluid with gastrointestinal contents. Although the dissection was performed without direct manipulation of the digestive tract, the anatomical proximity of digestive tissues in the abdomen may lead to the presence of nonhemolymph components in the extracted fluid. In this study, we use the term “extracellular fluid” in an operational sense, referring to the liquid recovered by passive centrifugation from intact body segments, without asserting biochemical exclusivity. Future versions of this method may include improved segmental dissection, targeted exclusion of digestive tissues, or biochemical markers to verify fluid origin.

### Practical applications

The methodology presented has direct applications in ecotoxicology, enabling the evaluation of contaminants, such as neonicotinoids (Brandt et al. 2016; Williamson et al. 2014; Tosi et al. 2017; Odemer et al. 2023) differentially affect the body regions of bees. Previous studies have shown that the accumulation of pesticides and their metabolic impact are not homogeneous in insects (Gonzalez et al. 2022). The segmented extraction of extracellular fluid proposed here provides a more precise approach to identifying patterns of accumulation and regional physiological effects.

Additionally, the optical characterization via UV-Vis spectroscopy demonstrates its utility as a noninvasive method for detecting biochemical differences in extracellular fluid. The presence of specific absorbance maxima in each segment suggests variability in the concentration of proteins and other metabolites (Chen et al. 2021; Chang et al. 2023; Wang et al. 2021), which could serve as biomarkers of physiological stress in response to environmental factors (Chang et al. 2023).

The absorbance spectra obtained from each segment suggest qualitative and quantitative differences in the composition of the extracellular fluid. The higher absorbance values observed in the abdominal samples may reflect a greater concentration of solutes, such as proteins, metabolic byproducts, or nitrogenous waste, which are more likely to accumulate in the storage and excretory regions of the insect. In contrast, the thoracic and head segments—dominated by muscle and neural tissue, respectively—may contain lower solute concentrations and different biochemical profiles. The segment-specific spectral peaks and differences in overall absorbance intensities support the presence of localized biochemical variation, which could be explored in future studies using targeted molecular or proteomic analyses.

This study also introduces a quantitative methodology for analyzing color changes in Benedict's reagent tests, a technique traditionally limited to qualitative assessments. The use of Fiji (ImageJ) software (Schindelin et al. 2012) enabled the extraction of RGB values from digital images, allowing for precise quantification of color differences among samples. This approach revealed that while thorax and abdomen samples appeared visually similar, significant differences were observed in their chromatic component values; this methodology not only enhances the sensitivity of the test, but also provides a reproducible and objective analysis, which is valuable for biochemical studies requiring the evaluation of subtle variations in sugar concentrations or other metabolic compounds.

The differences in coloration observed in the Benedict's reagent test likely reflect variations in the presence and concentration of reducing sugars among body segments. The more intense reddish hues in the head samples suggest a higher content of glucose or other reactive sugars, possibly related to neural tissue metabolism or localized storage. Thorax and abdomen samples displayed lower intensities, which may correspond to reduced sugar content or the presence of less reactive compounds. While this experiment did not determine the exact identity of the sugars involved, the RGB quantification supports segment-specific biochemical variation. Future work could integrate chromatographic or enzymatic techniques to more precisely characterize the carbohydrate profile of each segment.

The quantification of colors using digital tools represents a methodological advancement that can be applied to other traditional colorimetric tests, enabling the detection of subtle differences that might be overlooked in purely visual analyses. This precision is particularly relevant in comparative physiology studies and ecotoxicological research, where small changes in metabolites could be linked to environmental stress factors.

### Study limitations

Despite its advantages, the technique has certain limitations. The use of distilled water as a flow-facilitating agent may dilute specific metabolites present in the extracellular fluid, which should be considered when interpreting biochemical results. Additionally, the small size of samples obtained from smaller segments, such as the head, can pose challenges for studies requiring larger sample volumes.

Another consideration relates to the use of a fixed number of bees rather than a standardized segment mass during centrifugation. To address this, Experiment 1 provided a detailed characterization of body segment mass and water content, establishing a solid reference framework. This dataset enabled estimation of the mass applied in each extraction procedure, allowing for the inference of centrifugal forces applied to each segment type (see Fig. 2). By incorporating this information into the design and interpretation of Experiments 2–4, we ensured that differences in mechanical forces across segments were taken into account, thereby reinforcing the validity of comparative analyses.

In Experiments 3 and 4, the segment-specific samples were obtained using a fixed number of bees, as in previous steps. Although this approach introduced variations in total segment mass and, consequently, in the absolute centrifugal force, Experiment 1 provided reference values for segment masses and water content, which allowed contextual interpretation of the results.

In the case of UV-Vis spectroscopy, the absorbance values are indeed influenced by the concentration of solutes, which in turn can be affected by the volume of fluid extracted. We acknowledge that differences in segment mass and fluid yield may have contributed to the intensity of absorbance peaks. However, the segment-specific absorbance patterns, such as the position of maxima and the relative spectral profiles, reflect compositional differences that are independent of total fluid volume.

Similarly, RGB analysis from Benedict's reagent reactions focused on relative chromatic patterns rather than absolute color intensity. The consistency of these patterns across replicates supports their physiological relevance. Overall, the use of moderate centrifugation (2000 RCF for 4 min) and standardized imaging conditions helped minimize technical variability, allowing us to compare segmental biochemical signatures with confidence.

### Future perspectives

The findings of this study contribute to future research focusing on the molecular composition of segmented extracellular fluid using advanced techniques such as mass spectrometry or liquid chromatography. Additionally, the relationship between the composition of extracellular fluid and the physiological performance of bees under stress conditions, such as exposure to contaminants or environmental changes, represents a priority area of interest.

Finally, the proposed approach could be adapted to other animal models, enabling comparisons across different insect species and their responses to specific environmental factors; this regional analysis capability provides a powerful tool for studies in comparative physiology and pollinator conservation.

## Conclusion

This study developed and validated an efficient and reproducible methodology for the segmented extraction of extracellular fluid in *Apis mellifera* bees by using controlled centrifugation. The results demonstrated significant differences in the volume of fluid recovered, its optical properties, and the presence of reducing sugars among body segments, with the abdomen being the region with the highest extraction capacity. The spectroscopic and biochemical characterization of the samples highlighted the importance of analyzing each segment independently to obtain more precise information about the physiology and metabolism of bees.

This methodology represents a robust tool for physiological, metabolic, and ecotoxicological studies, with applications in assessing environmental impacts and pollinator health. In the future, optimizing the method and integrating it with advanced analytical techniques will allow for a deeper exploration of the molecular composition of extracellular fluid and its relationship with environmental and stress-related factors.
